# RNA elongation by respiratory syncytial virus polymerase is calibrated by conserved region V

**DOI:** 10.1371/journal.ppat.1006803

**Published:** 2017-12-27

**Authors:** Molly R. Braun, Laure R. Deflubé, Sarah L. Noton, Michael E. Mawhorter, Chadene Z. Tremaglio, Rachel Fearns

**Affiliations:** Department of Microbiology, Boston University School of Medicine, Boston, MA, United States of America; Harvard Medical School, UNITED STATES

## Abstract

The large polymerase subunit (L) of non-segmented negative strand RNA viruses transcribes viral mRNAs and replicates the viral genome. Studies with VSV have shown that conserved region V (CRV) of the L protein is part of the capping domain. However, CRV folds over and protrudes into the polymerization domain, suggesting that it might also have a role in RNA synthesis. In this study, the role of respiratory syncytial virus (RSV) CRV was evaluated using single amino acid substitutions and a small molecule inhibitor called BI-D. Effects were analyzed using cell-based minigenome and *in vitro* biochemical assays. Several amino acid substitutions inhibited production of capped, full-length mRNA and instead resulted in accumulation of short transcripts of approximately 40 nucleotides in length, confirming that RSV CRV has a role in capping. In addition, all six variants tested were either partially or completely defective in RNA replication. This was due to an inability of the polymerase to efficiently elongate the RNA within the promoter region. BI-D also inhibited transcription and replication. In this case, polymerase elongation activity within the promoter region was enhanced, such that the small RNA transcribed from the promoter was not released and instead was elongated past the first gene start signal. This was accompanied by a decrease in mRNA initiation at the first gene start signal and accumulation of aberrant RNAs of varying length. Thus, in addition to its function in mRNA capping, conserved region V modulates the elongation properties of the polymerase to enable productive transcription and replication to occur.

## Author summary

Respiratory syncytial virus (RSV) is a leading cause of respiratory illness in infants, elderly, and immunocompromised individuals, yet treatment is limited to supportive medical care. The large polymerase protein (L) of RSV is essential for transcribing viral mRNAs and replicating the genome, and is an attractive target for antiviral intervention. Previous studies from related viruses have shown that a region of the L protein co-transcriptionally adds a cap to nascent mRNAs. In this study, we showed that perturbation of this region of the RSV L protein resulted in capping failure, as expected. In addition, we found that this region altered how efficiently the polymerase could elongate the RNA, impairing recognition of essential signals. Thus, this region of the RSV L protein has more than one function during viral multiplication, making it a particularly appealing target for antiviral development.

## Introduction

RSV is a major cause of respiratory disease in infants, immunosuppressed individuals and the elderly. Currently there is no vaccine and the only means to mitigate the infection is by prophylactic administration of antibodies or ribavirin treatment in select cases [1]. Thus, there is a need for novel antivirals against the virus. RSV is a member of the Family *Pneumoviridae* in the Order *Mononegavirales*, the non-segmented, negative strand RNA viruses (nsNSVs) [2]. Like other viruses in the order, the RSV genome is transcribed and replicated by the viral RNA-dependent RNA polymerase. Because it is essential for viral multiplication, and possesses enzymatic properties, the polymerase is considered a highly promising target for antiviral drug development [3, 4].

The core RSV polymerase is comprised of a complex of two proteins: the 250 kDa large polymerase subunit (L), which contains the enzymatic domains involved in transcription and replication, and the 27 kDa phosphoprotein (P), which is an essential co-factor [5, 6]. To perform mRNA transcription, the polymerase initiates RNA synthesis from position 3 (+3) of the *leader* (*le*) *promoter* at the 3' end of the genome. This results in synthesis of a small le RNA [7]. This RNA is shorter than the 44 nt *le promoter* region, but heterogeneous in length, ranging from approximately 20–30 nt, with the dominant length being approximately 25 nt. This heterogeneity suggests that the polymerase does not release the RNA in response to a specific signal, but is relatively non-processive in the promoter region and releases the RNA after it has moved away from the core promoter. The polymerase then scans to the first gene at position 45 and transcribes the genome by responding to *cis*-acting signals that flank each of the genes [8]. A *gene start* (*gs*) signal directs the polymerase to initiate mRNA synthesis, and a *gene end* (*ge*) signal causes the polymerase to polyadenylate and release the mRNA [9–11]. During transcription, an elongation factor, M2-1, is required for synthesis of full-length mRNAs, particularly from the longer genes [12–14]. Replication of the genome involves production of uncapped, encapsidated antigenome and genome RNAs. Like mRNA transcription, RNA replication is initiated at a promoter at the 3' end of the *le* region [15–17]. In this case, the polymerase initiates RNA synthesis opposite position 1 of the promoter (+1) and synthesizes a full-length antigenome RNA [18–20]. The antigenome contains a *trailer* (*tr*) *promoter* region at its 3' end (the complement of the 5' terminal trailer region), which signals the polymerase to synthesize genome RNA from position 1 and a small tr RNA from position 3 [19, 21]. Replicative RNAs are encapsidated with nucleoprotein (N) as they are synthesized, which is thought to allow the polymerase to efficiently elongate the RNA to the end of the template [7, 8, 17, 22, 23]. Concurrent encapsidation might also be required to enable the polymerase to avoid releasing the RNA after ~ 25 nt in the promoter regions [8].

**Fig 1:**
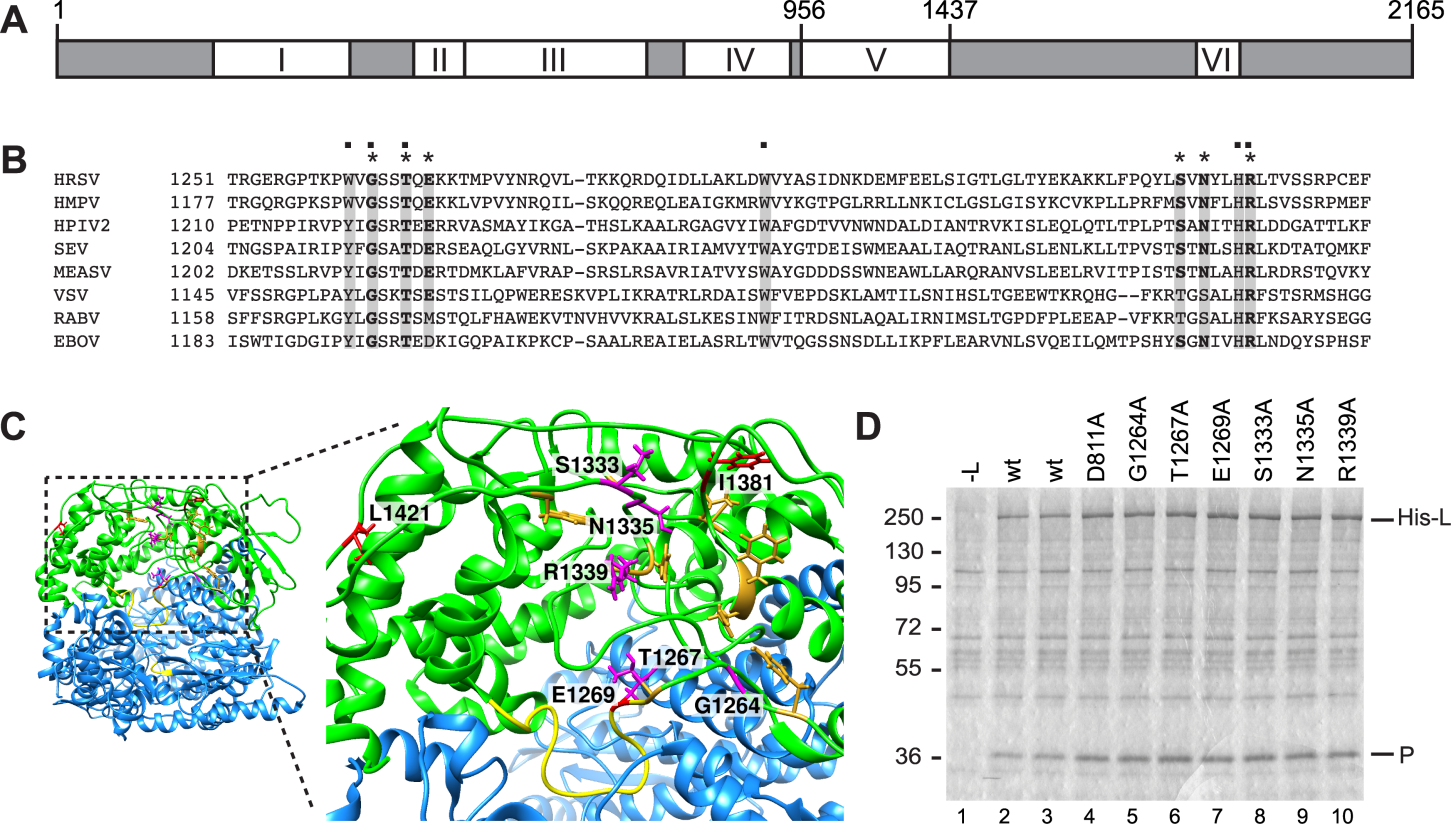
Organization of the L protein. (A) Schematic diagram showing the locations of CRI to VI within the RSV L protein, with the amino acid residue positions at the boundaries of CRV indicated. (B) Alignment of amino acids within CRV of L that have been shown to affect capping in VSV. L amino acid sequences were aligned with MegAlign software (DNASTAR, Lasergene) using the CLUSTAL W method, and conserved amino acids were highlighted with BOXSHADE. Conserved residues corresponding to those that are essential for VSV capping are indicated with a dot. Amino acids substituted in this study are indicated with asterisks. The L protein accession numbers are as follows: RSV (M75730); HMPV (Human metapneumovirus, YP_012613.1), HPIV2 (Human parainfluenza virus 2, NP_598406.1), SEV (Sendai virus, NP_056879.1), MEASV (Measles virus strain Schwarz, ACN50062.1), VSV (Vesicular stomatitis Indiana virus, AAA48441.1), RABV (Rabies virus strain SAD B19-1^st^, ACF94307.1), EBOV (Zaire Ebolavirus, NP_066251.1). (C) Structure of the polymerization and capping domains of VSV (colored blue and green, respectively), with CRV shown as a zoomed in image. VSV residues that are required for capping are shown in gold, and the putative priming loop and polymerization GDN motif are shown in yellow. Amino acid residues that were substituted in this study and BI-D resistance sites were mapped onto the VSV L structure [34] and are shown in magenta and red, respectively. The image was prepared using UCSF Chimera [53]. (D) Analysis of the expression levels of mutant his-tagged L proteins and their ability to bind to P protein. Colloidal blue stained SDS-polyacrylamide gel showing His-L-P complexes isolated by Ni-NTA affinity chromatography. Lane 1 is a negative control in which cells were transfected with a P expression plasmid, but not L.

In addition to their polymerization activity, nsNSV polymerases are responsible for adding a methylated cap to the mRNA [24–26]. Studies with members of the *Rhabdoviridae* have shown that their polymerases cap viral mRNAs by an unusual RNA:GDP polyribonucleotidyltransferase (PRNTase) activity, rather than by a guanylyltransferase reaction [26–29]. This activity has been mapped to a region of the polymerase called conserved region V (CRV; Fig 1A). Key residues within CRV are conserved across the nsNSVs (Fig 1B), indicating that this capping mechanism is likely conserved throughout the order [24, 26, 27, 30]. Capping occurs co-transcriptionally, presumably when the mRNA has reached a sufficient length to be extruded from the polymerization active site; in the case of vesicular stomatitis virus (VSV), this happens when the mRNA has reached 31 nucleotides [31]. Mutagenesis studies of the VSV capping domain have also shown that cap addition is required for efficient polymerase elongation during mRNA transcription, with capping failure correlating with production of transcripts of ~40 nt in length [24, 32, 33]. These findings suggest there is a checkpoint during mRNA synthesis to only allow elongation of successfully capped mRNA transcripts.

The VSV polymerase structure suggests that in addition to its defined role in capping, CRV might also affect RNA synthesis. In a pre-initiation form of the polymerase, CRV resides in a lobe that is folded over the polymerization domain, and a putative priming loop protrudes into the active site [34] (Fig 1C). A hint that CRV might play a role in RNA synthesis also comes from analysis of an RSV small molecule polymerase inhibitor called BI-D. Resistance to BI-D maps to CRV suggesting that it binds this domain [25] (Fig 1C). Experiments using an *in vitro* polymerization assay showed that BI-D enabled the RSV polymerase to reach the end of an oligonucleotide template more efficiently than in the absence of compound [35]. Based on these observations, we hypothesized that CRV might have other roles in transcription and replication beyond mRNA capping. In this study, we perturbed CRV using alanine substitutions or BI-D to determine 1) if CRV is involved in RSV mRNA capping, and 2) if CRV plays an additional role in RNA synthesis, unrelated to its role in mRNA capping. The data obtained show that CRV does affect capping, as expected, but that in addition, functions to regulate the elongation step of RNA synthesis. These two properties could be separated by single amino acid substitutions in CRV indicating that they are distinct. Moreover, the data indicate that the elongation properties of the polymerase must be appropriately calibrated for it to be able to perform transcription and replication.

## Results

### Introduction of substitutions into conserved region V of the RSV L protein

To tease apart the role of CRV during RSV transcription and genome replication, single amino acid alanine substitutions were introduced into T7-expressed L protein so that their effects could be monitored in a cell-based minigenome system. The substituted residues included G1264, T1267, E1269, S1333, N1335 and R1339, which lie within, or proximal to, the pocket defined by the polymerase variants that inhibited capping in VSV, and where BI-D resistance variations could be mapped (E1269 is one of the BI-D resistance sites; Fig 1C). As a control, a substitution was also introduced into conserved region III of L (D811), which is within the RNA synthesis catalytic domain [6, 36, 37]. Prior to performing functional analysis of the variant L proteins, we confirmed that the substitutions did not inhibit expression of the L protein. Because there was no effective antibody available against L protein, hexahistidine tagged versions of wt and variant L proteins were constructed. L requires co-expression of P to be efficiently expressed [6, 38] and so the RSV L and P proteins were co-expressed from T7 plasmids in HEp-2 cells. Western blot analysis gave only a very weak and inconsistent signal for L, likely due to poor transfer of the protein because of its large size. Therefore, as an alternative approach, L-P complexes were isolated by affinity binding to Ni-NTA agarose beads and examined by SDS-PAGE and colloidal blue staining (Fig 1D). This analysis showed that all L protein variants were expressed at a similar level as the wt L protein and retained their ability to bind P.

### Some conserved region V substitutions inhibited mRNA transcription

**Fig 2:**
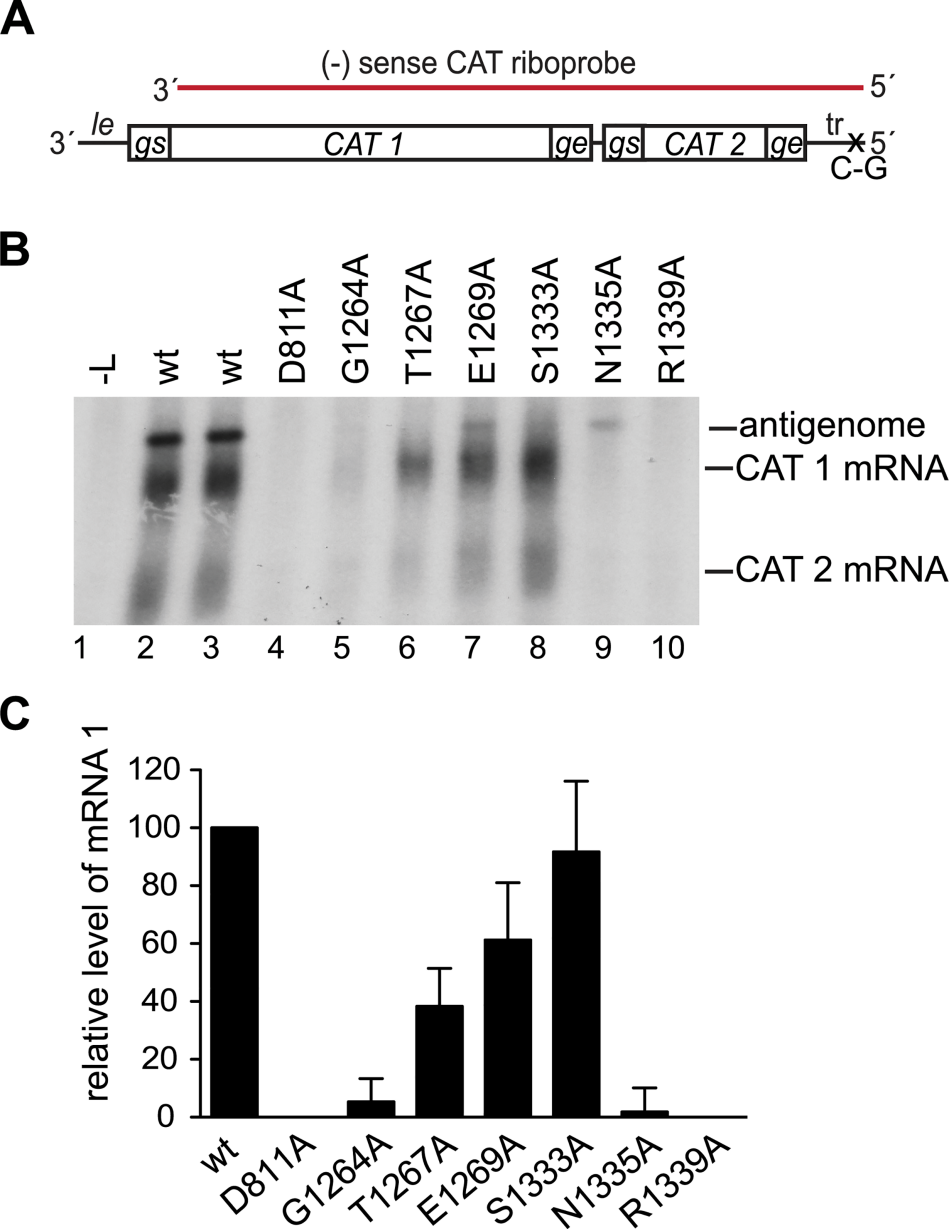
Substitutions in CRV inhibited transcription. (A) Schematic diagram (not to scale) of the minigenome used to analyze mRNA transcription. The total minigenome length is 970 nt, consisting of a 44 nt *leader (le) promoter* region, 580 nt CAT 1 gene, 1 nt intergenic region, 190 nt CAT 2 gene, and 155 nt trailer (tr) region. The minigenome was limited to the antigenome step of RNA replication by a single nucleotide substitution at position 2 relative to the 5' end of the tr region. The region contained in the CAT riboprobe is indicated in red. (B) Northern blot analysis of positive-sense RNA produced from the minigenome by wt and variant L proteins detected with the CAT riboprobe. The positions of the antigenome, and CAT 1 and 2 mRNAs are indicated. Lane 1 is a negative control of RNA from cells in which L plasmid was omitted from the transfection. (C) Levels of CAT 1 mRNA generated by the variant L proteins as determined by quantification of replicates of the experiment shown in panel B. The data are normalized to the mean of the two wt samples in each experiment at 100%. The bars show the means and standard deviations of three independent experiments.

The L variants were then analyzed in a minigenome assay [39] to examine the effects of the substitutions on RSV transcription and replication. The minigenome contained the *le promoter* at its 3' end and trailer sequence at its 5' end. The ten naturally occurring RSV genes were replaced with two genes containing different fragments of chloramphenicol acetyl transferase (CAT) sequence, each flanked with RSV *gs* and *ge* sequences (Fig 2A). To uncouple minigenome transcription from replication and allow the two processes to be examined as independent events, the 5' terminal trailer region was modified with a substitution at position 2 relative to its 5' end. This inactivated the *tr promoter* at the 3' end of the antigenome, preventing it from acting as a template for genome synthesis and thus limiting minigenome replication to the antigenome synthesis step, as described previously [16]. RSV RNA synthesis was reconstituted intracellularly by transfecting BSR-T7 cells with plasmids expressing the minigenome RNA and RSV N, P, M2-1, and L proteins. For this, and all following experiments using the minigenome system, BSR-T7 cells were used to supply T7 RNA polymerase, instead of MVA-T7, to avoid the possibility of artifacts arising from the capping enzyme activity of vaccinia virus. For these experiments, untagged L constructs were used, as the tag impaired polymerase activity to some extent. The products generated by the L variants were detected by Northern blot analysis using a negative sense riboprobe that would detect both mRNAs and the antigenome (Fig 2A). In cells transfected with wt L protein, three major RNA species accumulated, representing encapsidated antigenome, and polyadenylated CAT 1 and CAT 2 mRNAs (Fig 2B, lanes 2 and 3) [12]. The identities of these bands have been determined previously by manipulating *gs* and *ge* signals, and using specific probes (e.g. [13, 17]). Analysis of the L variants showed that the S1333A variant generated a relatively high level of mRNA, and the T1267A and E1269A variants showed moderate levels of mRNA production, as compared to the wt L protein (Fig 2B and 2C). The mRNAs produced by these variant polymerases were diffuse in nature, similar to those produced by the wt polymerase, indicating that they were polyadenylated. In contrast, the G1264A, N1335A, and R1339A polymerases yielded significantly reduced levels of mRNA compared to wt (p <0.05). Thus, several amino acid substitutions inhibited transcription.

### Transcription defects correlated with lack of cap addition

**Fig 3:**
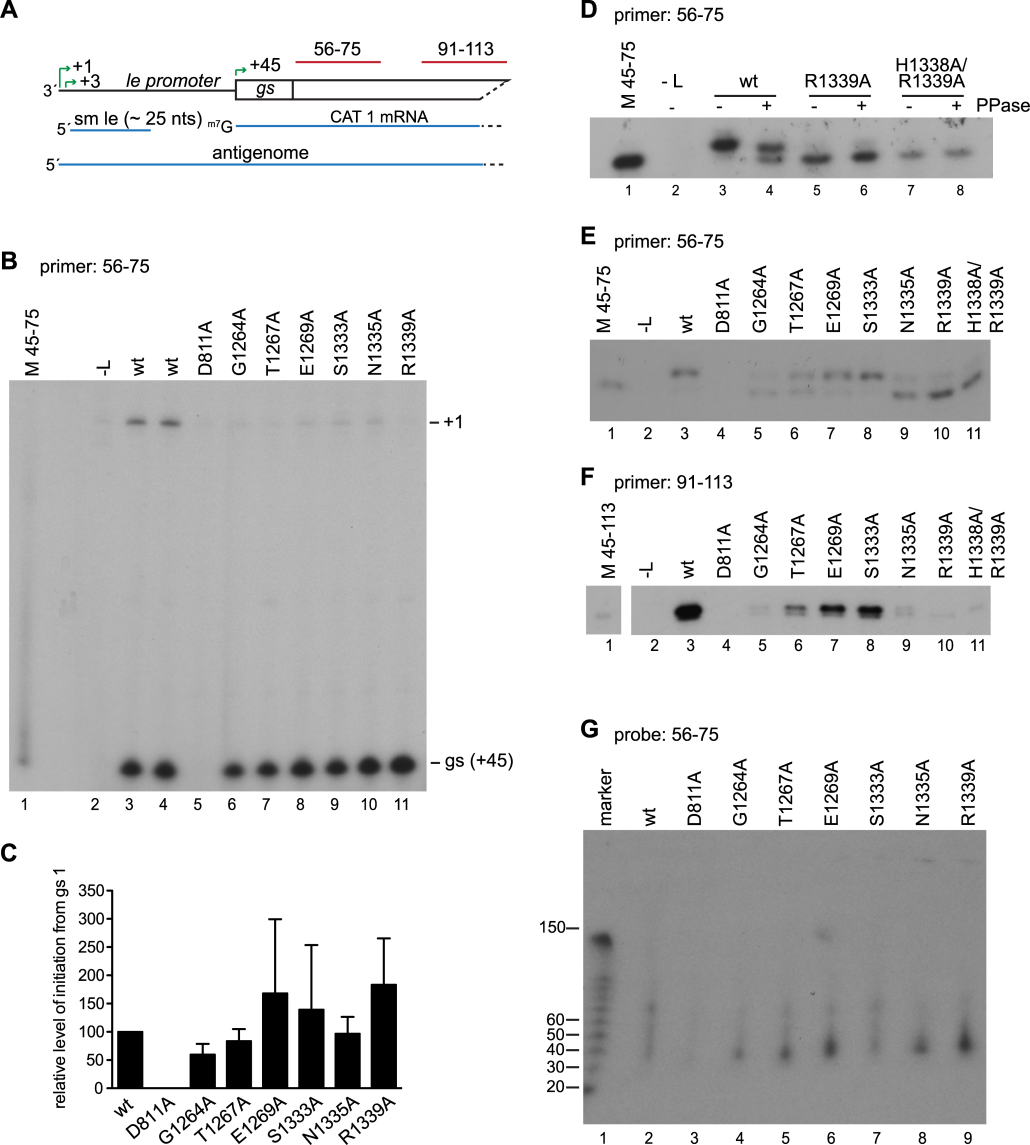
CRV substitutions inhibited mRNA capping. (A) Schematic diagram of the 3' end of the minigenome. The initiation sites within the *le promoter* (+1, +3) and at the first *gs* (+45) are indicated with green arrows, and the positive-sense small le (sm le), CAT 1 mRNA, and antigenome RNAs that are produced from this region of the minigenome are shown below in blue. The regions contained within the negative sense 56–75 and 91–113 primers and probes are indicated in red. (B) Primer extension analysis of RNA generated by wt and variant L proteins using the 56–75 primer and Sensiscript reverse transcriptase. Lane 1 is a marker prepared by end-labeling a 31 nt DNA oligonucleotide corresponding in sequence to the primer extension product representing initiation at the first *gs* signal (M 45–75). (C) Quantification of RNA initiated at the *gs* signal based on replicates of the experiments shown in panel B. The data are normalized to a mean of the two wt samples included in each experiment at 100% and the bars show the mean and standard deviation for three independent experiments. (D) Primer extension analysis of the RNA generated by wt and the R1339A and H1338A/R1339A variant polymerases using the 56–75 primer and Thermoscript reverse transcriptase. RNAs were treated with buffer only, or pyrophosphatase in buffer, as indicated by -/+ symbols. (E and F) Primer extension analysis of RNA generated by wt and variant L proteins, performed with Thermoscript reverse transcriptase with either the 56–75 (E), or 91–113 primer (F). (G) Northern blot analysis of small RNA transcripts produced from the minigenome. The RNA was migrated on a 6% urea-acrylamide gel and detected with an end-labeled 56–75 oligonucleotide probe.

Given that CRV is known to be involved in mRNA capping, the block to transcription was likely to be at the capping step. We have been unable to reconstitute efficient PRNTase activity *in vitro* using purified polymerase, as has been described for VSV, Chandipura and rabies viruses [24, 26, 28, 40] to confirm this directly. This might be because the four guanosines that lie at the 5' end of RSV mRNAs have the potential to form G-quadruplexes, preventing oligonucleotides containing this sequence from being bound as a substrate in a *trans*-capping assay. Therefore, we used primer extension analysis to examine if the mRNAs produced by the variant polymerases were capped. As described in the Introduction, the cap is added co-transcriptionally after the mRNA has reached a given length. If the substitutions inhibiting transcription were specifically targeting the PRNTase activity of the polymerase, it would be expected that the polymerase would retain its ability to initiate mRNA synthesis at the first *gs* signal at the beginning of the *CAT 1* gene. To determine if the variant L proteins were capable of mRNA initiation, the RNA samples were subjected to primer extension analysis using probe 56–75, which corresponds to nucleotides 56–75 of the minigenome and so hybridizes to nucleotides 12–31 at the 5' end of the CAT 1 mRNA transcript (Fig 3A). If RSV were similar to VSV, in which the cap is added when the transcript reaches 31 nucleotides in length [31], it would be expected that this primer would be able to bind to transcripts irrespective of whether they were capped or uncapped. Primer extension analysis was first performed using a reverse transcriptase that does not recognize the cap, which allowed accurate determination of initiation site selection. Analysis of RNA produced by the wt L protein revealed two bands, corresponding to antigenome initiated at position 1 of the genome, and RNA generated from the *gs* signal (position 45) (Fig 3B, compare lanes 3 and 4 with lanes 2 and 5). RNA initiated at the *gs* signal was also readily detected in reactions containing the L variants (Fig 3B, lanes 6–11). There was experimental variation in the levels of this RNA (Fig 3C), likely because some of these products were relatively unstable. Nonetheless, the data clearly showed that all variants were capable of initiating mRNA synthesis from the *gs* signal. Comparison of transcript length with a molecular weight marker that indicated the length of transcripts initiated at the beginning of the *gs* signal (position 45) also showed that the transcripts were initiated at the correct position (Fig 3B, compare lane 1 with lanes 3–11). Thus, the variant polymerases were able to initiate mRNA synthesis from the *gs* signal, with no significant difference from wt.

To determine if the mRNAs were capped, experiments were performed using a different reverse transcriptase capable of reverse transcribing the guanosine cap (Fig 3D–3F). To establish this assay, an additional control was used, in which a double alanine substitution was introduced into the HR motif (H1338/ R1339). In VSV, both residues are essential for PRNTase activity, and the histidine forms a covalent linkage to the mRNA substrate [29]. Reverse transcriptase products of RNA produced by the wt polymerase migrated as a doublet: a faint lower band migrated alongside the marker (representing uncapped RNA), and a dominant upper band migrated slightly more slowly (Fig 3D, lane 3). Treatment of the RNAs with pyrophosphatase to cleave the linkage between the guanosine cap and RNA resulted in a reduction in the level of the upper band, and an increase in the signal from the lower band (Fig 3D, lanes 3 and 4). This finding showed that the upper band corresponded to capped RNA (cap removal might have been incomplete because the minigenome mRNA was within a pool of total cellular mRNA, which would have competed for enzyme). In the case of the H1338A/ R1339A variant, the lower band was the only dominant band detected (Fig 3D, lane 7). Faint traces of slower migrating RNA could be detected, but this was unaffected by pyrophosphate cleavage (Fig 3D, lane 8) and might be due to low level stuttering of the reverse transcriptase on the repetitive sequence that lies at the 5' end of RSV mRNAs (5' GGGG). The R1339A variant had a similar phenotype as the H1338A/R1339A variant, demonstrating that this was defective in capping (Fig 3D, lanes 5 and 6). Analysis of the RNAs produced by the other variant polymerases showed that S1333A variant was efficient in capping, the T1267A and E1269A variants were somewhat less efficient, and the G1264A and N1335A variants capped with poor efficiency (Fig 3E). The RNAs were also examined with a primer that hybridized further downstream on the mRNA transcripts (primer 91–113), which binds at positions 47–69 from the 5' end of the mRNA. Although this primer detected products from the wt, T1267A, E1269A and S1333A variants, it could only detect very low levels of RNA from the G1264A, N1335A and R1339A variants (Fig 3F). This result shows a correlation between the ability of the polymerase to cap the RNA and to elongate it far enough to be detected by the 91–113 primer.

### Uncapped transcripts were aborted after approximately 40 nt

To examine the lengths of the RNA produced by the G1264A, N1335A and R1339A variants, the RNA samples were subjected to Northern blot analysis using gel electrophoresis conditions optimized for examining short RNAs and probed with the 56–75 oligonucleotide used for the primer extension analysis (Fig 3A and 3G). For wt L protein, there were only faint bands apparent under these electrophoresis conditions, suggesting that this protein generates predominantly full-length mRNA (Fig 3G, lane 2). A similar result was obtained for the S1333A variant, which was highly efficient for capping and full-length mRNA synthesis (Fig 3G, lane 7). In contrast, the other variants produced RNAs that were ~30–50 nt in length, with the dominant signal at ~40 nt. The quantities of the ~40 nt RNAs detected for the G1264A, T1267A, E1269A, N1335A and R1339A variants were somewhat inconsistent between experiments, likely because the small size of this RNA meant it was not efficiently retained on the membrane. However, it was consistently observed that the variants most defective in transcription produced a ~ 40 nt RNA, which was not produced by the wt or S1333A variants. Together the data in Figs 2 and 3 show that substitutions in CRV cause a defect in transcription that correlates with inhibition of capping. The data also suggest that capping is required for RNA to be elongated beyond ~ 40 nt, consistent with what has been shown for VSV.

### Substitutions in conserved region V inhibited RNA replication elongation within the promoter region

**Fig 4:**
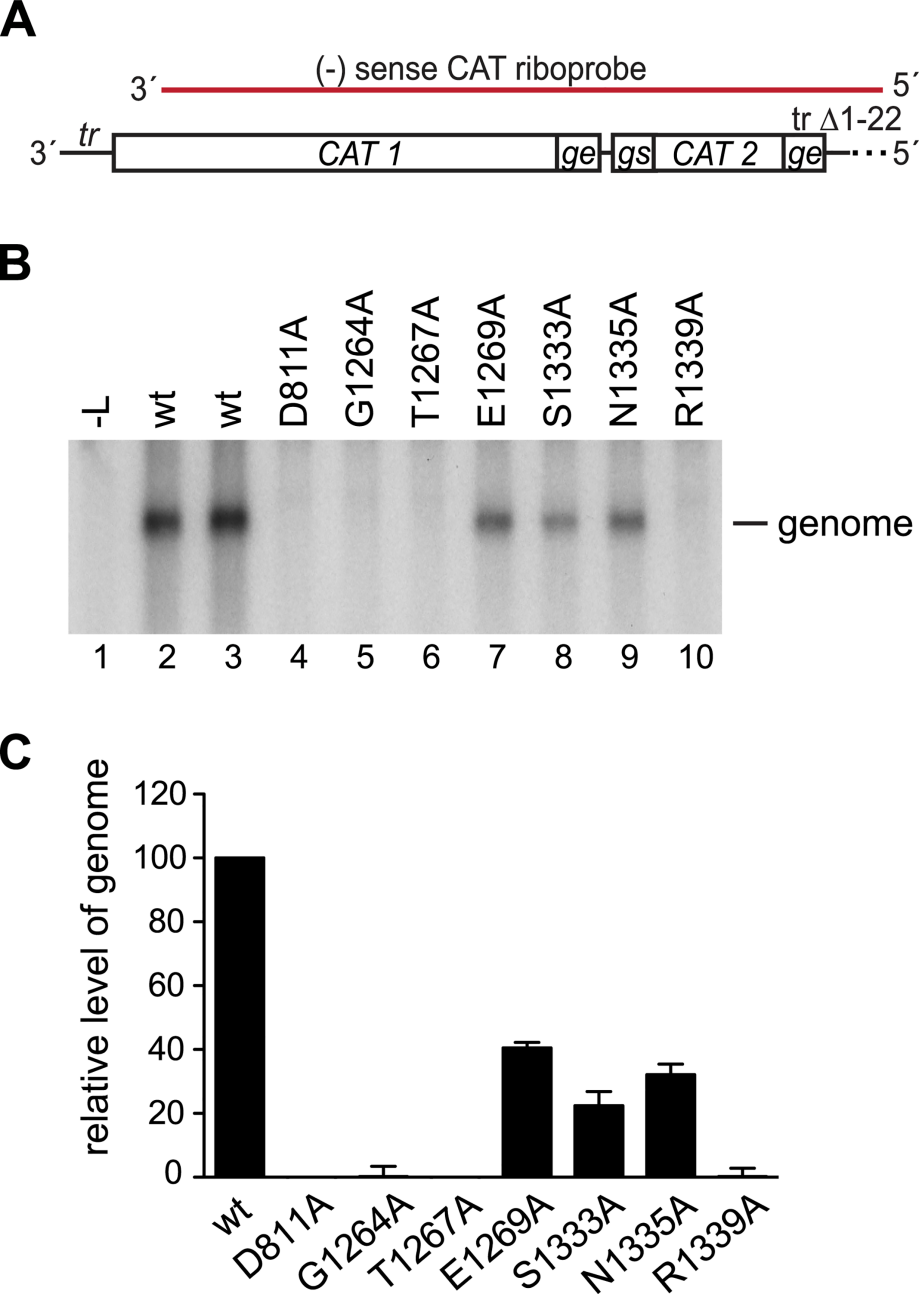
CRV substitutions inhibited RNA replication. (A) Schematic diagram (not to scale) of the minigenome template used to analyze RNA replication. The 3' end of the minigenome contained nucleotides 1–36 of the *tr promoter* directly adjoining CAT-specific sequence, and the 5' end had a 22 nucleotide deletion at the 5' terminus of the trailer region, indicated with a dotted line. The region contained within the riboprobe is indicated in red. (B) Northern blot analysis of replicative RNA (genome) produced from the minigenome by wt and variant L proteins, detected with the CAT-specific riboprobe. (C) Levels of genome generated by the variant L proteins as determined by quantification of replicates of the experiment shown in panel B, as described for Fig 2.

The data presented in Fig 2 show that, in addition to inhibiting transcription, some CRV variants were deficient in RNA replication, producing low to undetectable levels of antigenome. Given that replication products of nsNSVs are not capped, this finding shows that CRV has another role besides capping. To examine the effect of CRV substitutions on RNA replication specifically, the effects of the substitutions were analyzed using a replication-specific minigenome, in which the *le promoter* was replaced with nucleotides 1–36 of the *tr promoter* and the first *gs* signal was deleted (Fig 4A). The *tr* is a somewhat stronger replication promoter than the *le* [41], and the absence of a *gs* signal adjacent to the promoter prevents mRNA synthesis [11], allowing replication products to be measured without contamination with signal from polyadenylated monocistronic and readthrough mRNAs. Northern blot analysis showed that all the variants generated significantly lower levels of replication product (genome) than wt L protein (p <0.005 for each variant), with variants E1269A, S1333A, and N1335A generating replication product at a reduced level compared to wt (Fig 4B, lanes 7–9; 4C), and no product being detected for variants G1264A, T1267A, or R1339A (Fig 4B, lanes 5, 6, and 10; 4C). Thus, substitutions in CRV caused defects that affected replication in addition to transcription and the amino acid requirements for the two processes were not identical, with substitution of N1335 inhibiting transcription to a greater extent than replication, and the opposite being the case for T1267 (compare Figs 2 and 4).

**Fig 5:**
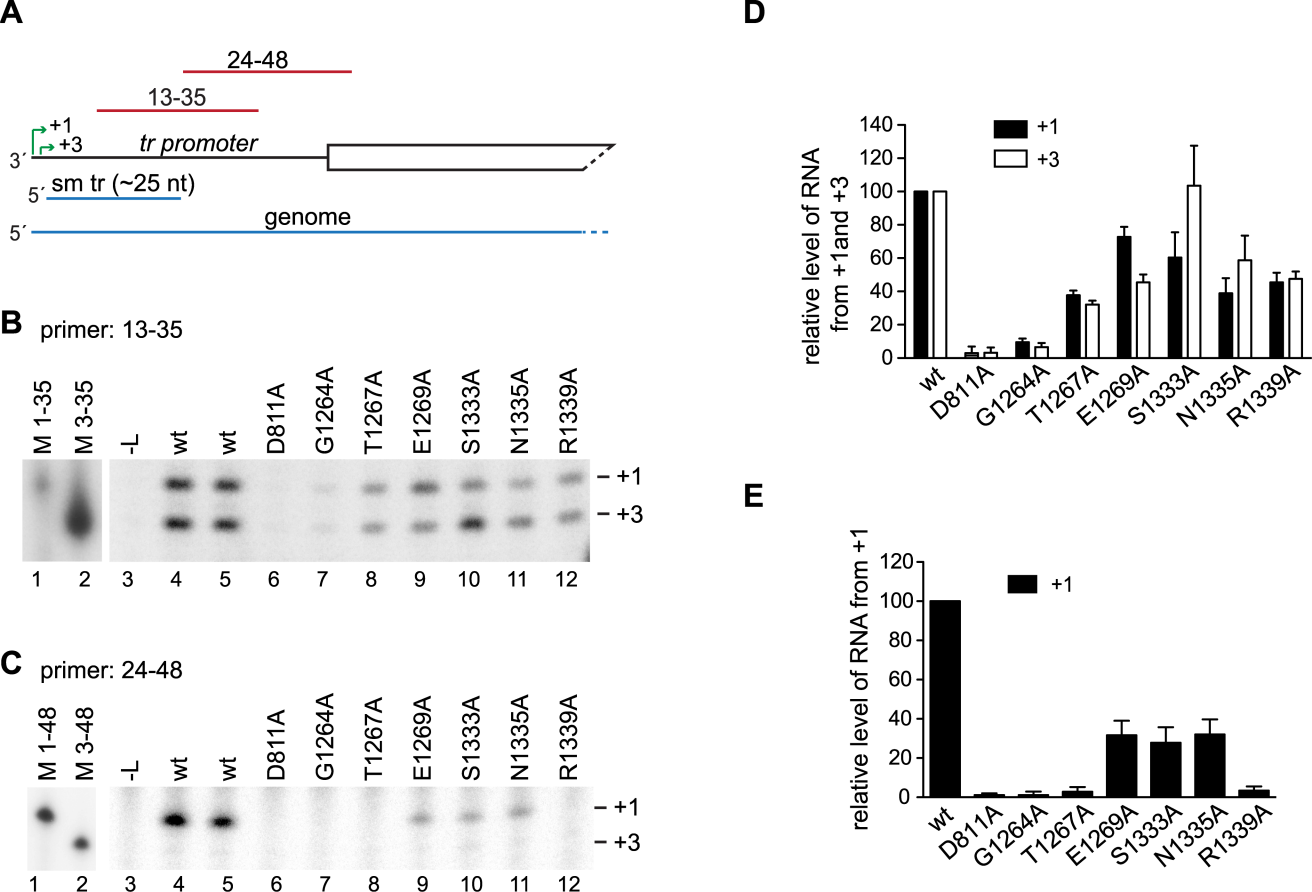
CRV substitutions inhibited the elongation step of replication. (A) Schematic diagram of the 3' end of the replication-specific minigenome. The two initiation sites in the *tr promoter* (+1, +3) are indicated with green arrows, and the small tr (sm tr) and genome RNA products are shown in blue. The regions contained within the 13–35 and 24–48 primers are indicated in red. (B, C) Primer extension analysis of RNA generated by wt and variant L proteins using the 13–35 and 24–48 primers, respectively. The position of the +1 and +3 initiation sites were determined by co-migration of end-labeled DNA oligonucleotides, corresponding in length and sequence to cDNAs representing initiation from those sites (Lanes 1 and 2). Lane 3 shows RNA from a control transfection lacking L expression plasmid. The white lines between lanes 2 and 3 indicate where empty lanes were excised. (D, E) Quantification of replicates of the experiments shown in panels B and C. Panel D shows levels of RNA initiated at +1 and +3, detected by primer extension with primer 13–35. Panel E shows levels of RNA initiated at +1 detected by primer 24–48. In D and E the data are normalized to a mean of the two wt samples included in each experiment at 100%. In panel D, the bars show the mean and range of two independent experiments. In panel E, the bars show the mean and standard deviation of three independent experiments.

RNA synthesis can be divided into different stages, including initiation and RNA elongation. Primer extension analysis was performed to narrow down which of these steps in RNA replication was affected by the substitutions. The RNA was analyzed using two primers that corresponded in sequence to nt 13–35 and 24–48 relative to the 3' end of the minigenome (Fig 5A). Analysis of the RNA generated by the wt L protein using primer 13–35 showed RNA initiated at the +1 and +3 sites of the *tr promoter* (Fig 5B, lanes 4 and 5), consistent with previous results [6, 21]. This primer also detected relatively high levels of RNA from +1 and +3 for each of the variants, except G1264A, for which only a very weak signal was obtained (Fig 5B, lanes 7–12, 5D). This shows that each of the variants, except G1264A, was capable of efficiently initiating RNA replication at +1 of the promoter and elongating the RNA far enough to be detected by the 13–35 primer. When RNA generated by the wt L protein was analyzed with primer 24–48, RNA initiated from +1 of the promoter could be efficiently detected, but RNA initiated from +3 was only barely detectable because most of this RNA is terminated at approximately 25 nt (Fig 5C, lanes 4 and 5). Analysis of the RNA generated by the variant L proteins showed that in contrast to what could be detected by the 13–35 primer, replication product initiated from +1 could only be detected for the three variants capable of generating full-length RNA replication products: E1269A, S1333A and N1335A, but not the other variants (Fig 5C and 5E). The levels of RNA detected for the E1269A, S1333A and N1335A variants were approximately 30% of wt levels, consistent with the levels of full-length RNA replication product detected by Northern blot analysis shown in Fig 4. One possible explanation why the 13–35 primer could bind RNA when the 24–48 primer could not, could be that the replicative RNA was degraded. However, primer extension analysis showed no evidence of replicative RNAs with heterogeneous termini, as would be expected if it were degraded by an endonuclease (see also Fig 3B), and it is difficult to rationalize why the 5' terminal fragments detected by the 13–35 primer would be protected if the RNA were degraded by a 3' to 5' exonuclease, given that there is no obvious RNA secondary structure in this region. Therefore, the fact that for each of the variants (except G1264A), the replication products could be detected by the 13–35 primer at a higher level relative to wt than by the 24–48 primer, indicates that substitutions in CRV inhibited elongation of replicative RNA within the promoter region.

### BI-D inhibited minigenome transcription and replication

**Fig 6:**
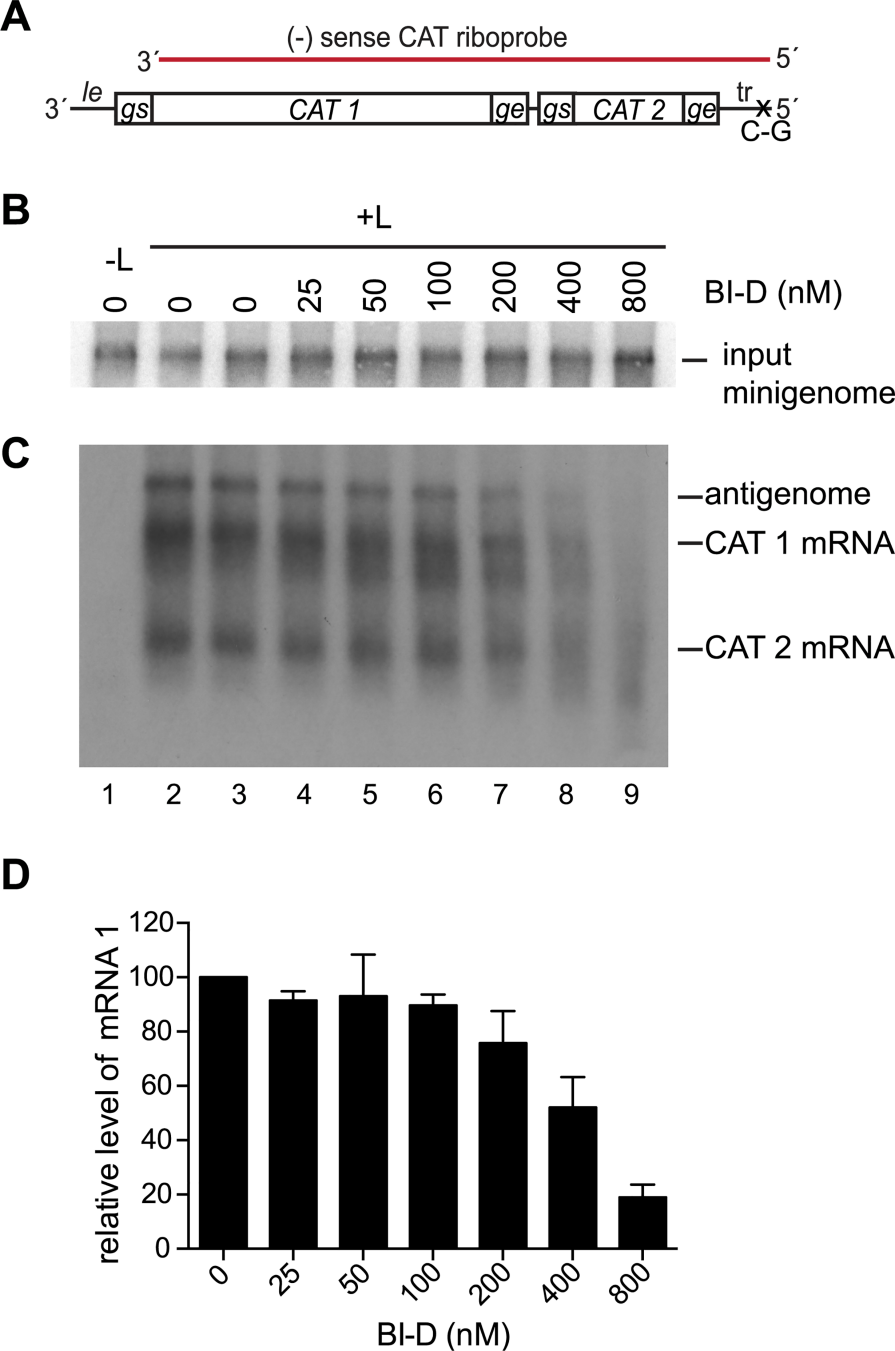
**BI-D inhibited transcription** (A) Schematic diagram of the transcription-competent minigenome as described in Fig 2A, with the regions contained within the CAT riboprobe indicated in red. (B) Northern blot analysis of input negative-sense minigenome RNA generated by T7 polymerase in the presence of varying concentrations of BI-D. (C) Northern blot analysis of positive-sense RNA produced from the minigenome, detected with the CAT riboprobe. (D) Quantification of CAT mRNA 1 where data are normalized to a mean of the two independent samples treated with DMSO included in each experiment, at 100%. The bars show the mean and range of two independent experiments.

As a complementary approach for examining the functions of CRV, we investigated how transcription and replication were affected by BI-D. As described in the Introduction section, BI-D is a small molecule inhibitor of the RSV polymerase. Resistance to BI-D maps to CRV (Fig 1C), implying that CRV is either the site where the compound binds the polymerase, or is a domain that it alters allosterically, making BI-D a useful tool for studying the activities of CRV. Cells were transfected with the transcription-competent minigenome used in Fig 2 (Fig 6A) and support plasmids, and incubated with varying concentrations of BI-D from the time of transfection until harvest. Northern blot analysis showed that the level of input minigenome template produced by T7 RNA polymerase was similar for each reaction, confirming that BI-D did not cause significant cytotoxicity at the concentrations used (Fig 6B). Northern blot analysis of the positive sense RNA products using the negative sense riboprobe showed that as the concentration of BI-D was increased, there was a decrease in the levels of full-length mRNA and antigenome, and an accumulation of smaller RNAs of heterogeneous size (Fig 6C, lanes 4–9, 6D). Thus, similarly to substitutions in CRV, BI-D inhibited transcription and replication.

### BI-D inhibited transcription by preventing release of the ~25 nt small le transcript

**Fig 7:**
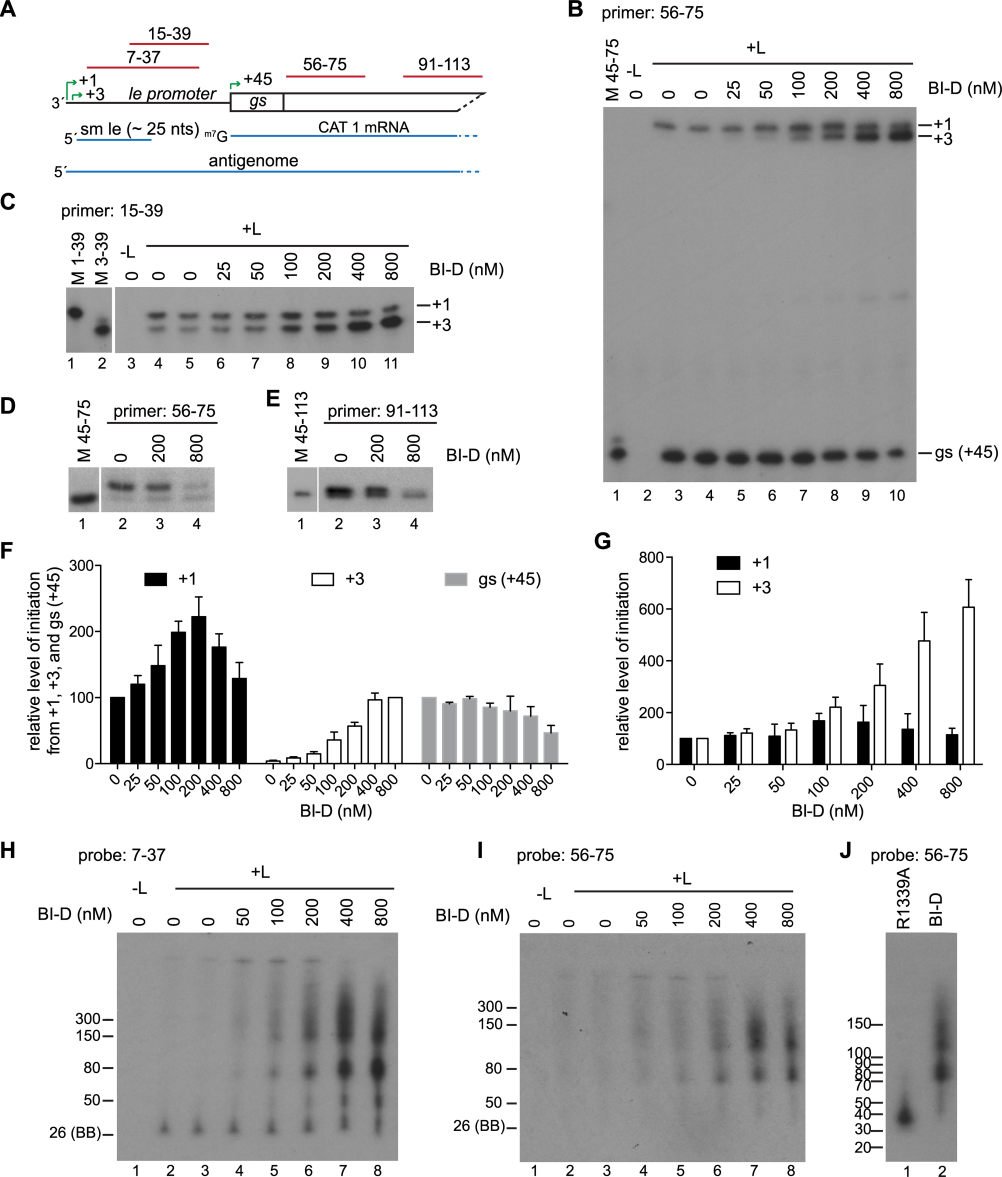
BI-D inhibited transcription by preventing release of small le RNA and caused accumulation of abortive RNAs. (A) Schematic diagram of the 3' end of the transcription-competent minigenome. The RNA products are shown in blue and the regions contained within the primers and probes are indicated in red. (B and C) Primer extension analysis of RNAs produced from the minigenome in the presence of varying concentrations of BI-D, using Sensiscript reverse transcriptase and primers 56–75 (B) and 15–39 (C). (D, E) Primer extension analysis of RNAs with Thermoscript reverse transcriptase and either the 56–75 (D) or 91–113 primer (E). In panels B, D, and E, lane 1 shows markers of oligonucleotides representing the primer extension products from the respective initiation sites; in panel C, lanes 1 and 2 show markers. Note that in panels C-E, the markers were migrated on the same gels as the adjacent samples, but a different exposure was required to visualize them. (F, G) Quantification of replicates of the experiments shown in panels B and C. Panel F shows levels of RNA initiated at +1, +3, and *gs* detected by primer 56–75. Panel G shows levels of RNA initiated at +1 and +3, detected by primer extension with primer 15–39. The +1 and *gs* products in panel F and the data in panel G were normalized to the—BI-D control in each experiment, which was set to 100%. In panel F, because the +3 products analyzed with primer 56–75 were at the level of background in the absence of BI-D, the +3 products were normalized to the value obtained with 800 nM BI-D, which was set to 100%. The bars show the mean and standard deviation of three independent experiments. (H, I) Northern blot analysis of small RNAs produced from the transcription-competent minigenomes by L protein in the absence or presence of BI-D. (J) Comparison of the sizes of abortive RNAs produced by a capping-deficient L variant (R1339) or by wt L protein in the presence of 800 nM BI-D. In H-J, RNA was migrated on 6% urea-acrylamide gels and detected with the indicated probe. The ladders are described in materials and methods. The 26 nt marker was based on the migration of the bromophenol blue (BB) dye on a 6% gel.

Primer extension analysis was performed using the 56–75 primer (Fig 7A) to examine how BI-D was inhibiting transcription. This analysis showed that as the concentration of BI-D was increased, there was a decrease in the level of RNA initiated at the *gs* signal (+45) (Fig 7B and 7F, gray bars). In parallel with this decrease, there was an appearance and increasing level of RNA initiated close to the antigenome initiation site (Fig 7B). The most likely explanation for the appearance of this additional band was that it represented RNA initiated at +3, but elongated beyond the end of the *le* region rather than being released after ~25 nt. To determine if this was the case, the RNA was analyzed using a primer that corresponded to nt 15–39 of the *le promoter* (Fig 7A and 7C). This analysis showed that the only initiation sites near the 3' end of the *le* region were at +1 and +3 (Fig 7C), confirming that the abnormal band detected with the 56–75 primer was initiated at position 3. At lower concentrations of BI-D (≤ 50 nM) there was no significant increase in +3 initiated RNA that could be detected by the 15–39 primer, however, there was a significant change in the level of +3 RNA that could be detected by the 56–75 primer, being completely undetectable in the absence of BI-D and clearly detectable, albeit as a faint band, at 25 and 50 nM BI-D (Fig 7, compare panel B, lanes 3–6 with panel C, lanes 4–7; panels F and G, white bars). This suggested that BI-D was causing an increase in elongation of the RNA initiated at +3, allowing it to become detectable by the downstream 56–75 primer. As the concentration of BI-D was increased, there was an increase in the level of +3 RNA that could be detected by the 15–39 primer. This could indicate an increase in initiation at the +3 site, which could contribute to the increased levels of this RNA that could be detected by the 56–75 primer, or it could be due to increased elongation of the RNA allowing it to bind more efficiently to the 15–39 primer.

To investigate these possibilities further, RNA samples were subjected to Northern blot analysis using gel electrophoresis conditions optimized for examining short RNAs and probed with an oligonucleotide that correlated in sequence to nucleotides 7–37 relative to the 3' end of the minigenome (Fig 7A) so that the lengths of RNAs initiated within the promoter region could be determined. In the absence of BI-D, the ~ 25 nt band, representing RNA initiated at +3 and released within the *le promoter* region was the only clearly detectable band. As the concentration of BI-D was increased, the levels of this band remained similar, but longer heterogeneous RNAs were detected (Fig 7H). The bands ≥ 70 nt could also be detected with the 56–75 probe, consistent with these being le-CAT 1 read through RNAs (Fig 7I). This analysis suggested that at least some of the RNA initiated at +3 was being elongated into the CAT 1 gene. Together, the primer extension and Northern blot data indicate that BI-D caused the polymerase to elongate the RNA beyond the promoter region, preventing efficient initiation at the first *gs* signal.

Previous studies with compound BI-A, an inhibitor in the same class as BI-D, had suggested that it could inhibit mRNA capping as it resulted in inhibition of full-length mRNA synthesis and instead caused production of abortive transcripts of < 50 nt [25]. To determine if BI-D inhibited capping, the RNA was also analyzed by primer extension analysis using a reverse transcriptase capable of detecting a cap, and primers 56–75 and 91–113, as described for Fig 3. This analysis showed that, compared to the untreated control, 200 nM BI-D caused no detectable difference in the ratio between the bands representing capped and uncapped RNA, indicating that this concentration did not inhibit capping activity. However, the ratio of bands was altered in reactions that had been treated with 800 nM BI-D, with the upper band of capped RNA being much less intense relative to the lower band of uncapped RNA (Fig 7D and 7E). These data show that BI-D could inhibit capping, in addition to affecting elongation within the promoter region. A direct comparison of RNA produced by the cap-defective R1339A variant L protein and by the wt L protein in the presence of 800 nM BI-D showed that the abortive RNAs that were produced were different in size (Fig 7J). Thus, these data show that BI-D inhibited capping, as expected, however, they also suggest that an additional effect was to cause abnormal elongation of the RNA.

### BI-D inhibited RNA replication from the *tr* promoter

**Fig 8:**
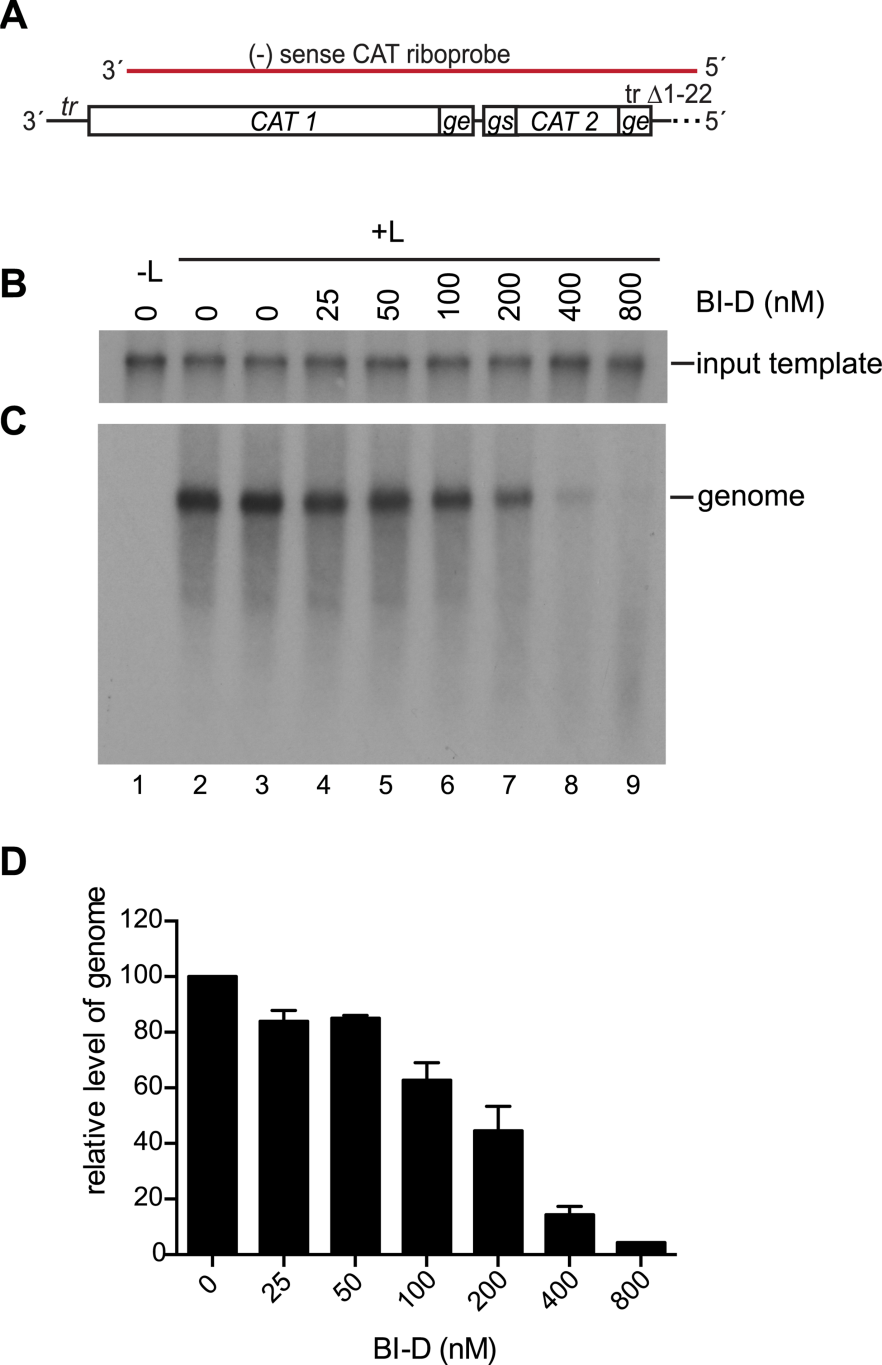
BI-D inhibited replication. (A) Schematic diagram of the 3' end of the replication-competent minigenome, with the regions contained within the riboprobe indicated in red. (B) Northern blot analysis of input minigenome generated by T7 polymerase during BI-D treatment. (C) Northern blot analysis of replicative RNA produced from the minigenome detected with the CAT riboprobe. (D) Quantification of genome RNA. The data are normalized to the mean of the two DMSO-only control samples in each experiment at 100%. The bars show the mean and standard deviation for three independent experiments, except for the 800 nM BI-D value, which is the mean of two experiments.

**Fig 9:**
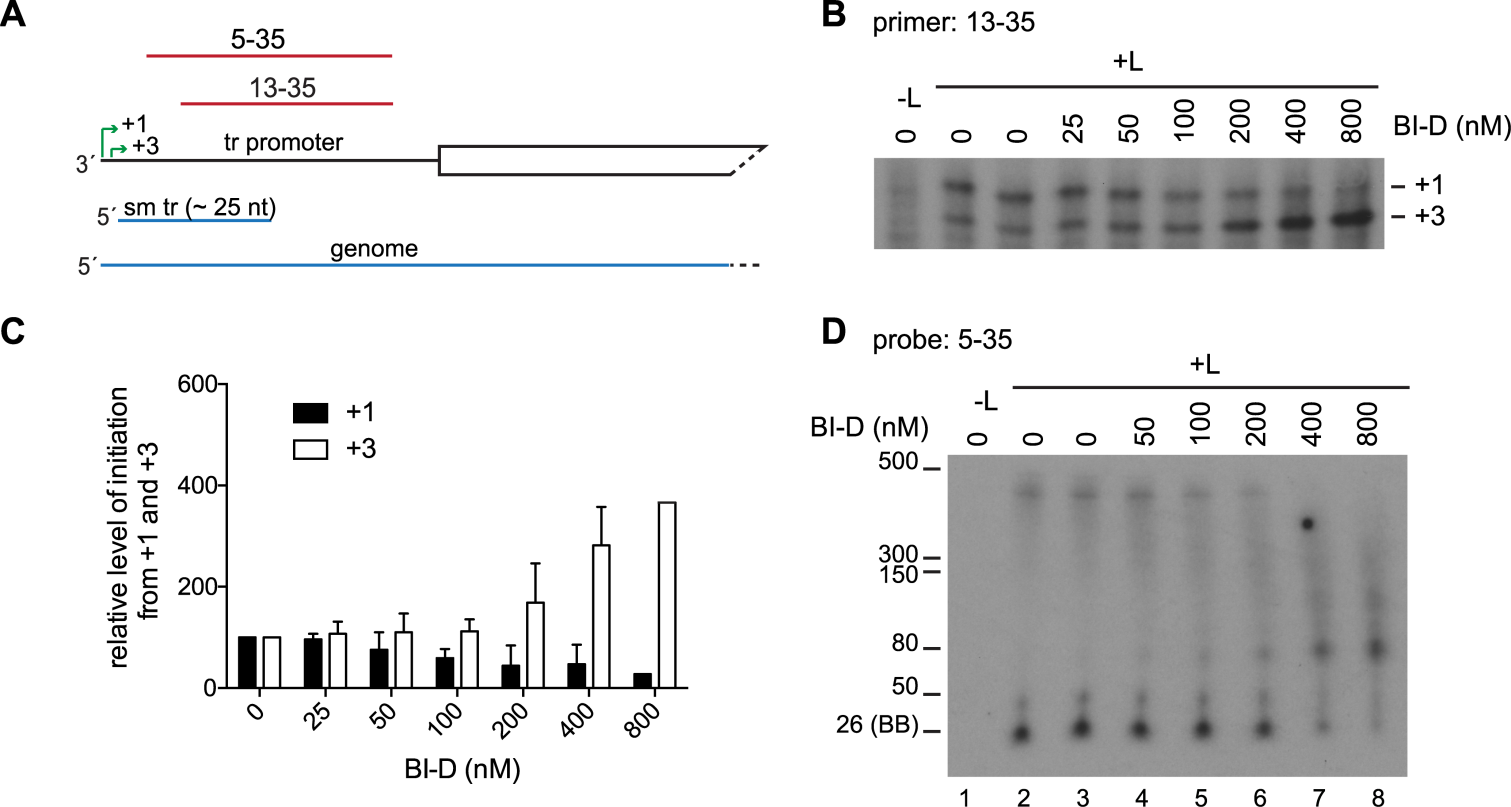
BI-D inhibited replication initiation and caused abnormal elongation within the promoter. (A) Schematic diagram of the 3' end of the replication-specific minigenome. The two initiation sites in the *tr promoter* (+1, +3) are indicated with green arrows, and the small tr (sm tr) and genome RNAs are shown in blue. The regions contained within the 5–35 and 13–35 primers and probes are indicated in red. (B) Primer extension analysis of RNAs produced from the minigenome; the positions of the +1 and +3 initiation sites were determined as described in Fig 5. (C) Quantification of replicates of the experiments shown in panel B. The data are normalized to the mean of the two DMSO-only control samples in each experiment at 100%. The bars show the mean and standard deviation for three independent experiments, except for the 800 nM BI-D value, which is the mean of two experiments. (D) Northern blot analysis of small RNA transcripts produced from the BI-D treated replication specific minigenome. The RNA was migrated on a 6% urea-acrylamide gel and detected with the 5–35 probe. The 26 nt marker was based on the migration of the bromophenol blue (BB) dye on a 6% gel.

To more clearly detect the effects of BI-D on replication, the inhibitor was tested using the replication-specific minigenome that lacked a *gs* signal after the promoter, as described in Fig 4 (Fig 8A). This analysis confirmed that BI-D did not affect synthesis of the input minigenome (Fig 8B) and showed that BI-D inhibited replication, and instead caused production of abortive RNAs (Fig 8C and 8D). Primer extension analysis was performed to examine RNAs initiated at positions 1 and 3 (Fig 9A and 9B). As was the case with the *le promoter*, there was an increase in the level of RNA initiated from +3, but in experiments with the *tr promoter*, there was a more pronounced decrease in the level of RNA that could be detected from +1 (Fig 9C). Analysis of the small *tr promoter* specific RNA by Northern blotting with a probe corresponding to nt 5–35 of *tr* showed a similar pattern of RNA products as those synthesized from the *le promoter* (Fig 9D). In this case, as the concentration of BI-D was increased, there was a clear decrease in the ~25 nt RNA and an increase in the levels of longer RNAs, again suggesting that BI-D was causing an increase in elongation of the RNA initiated at +3.

### BI-D inhibitor prevented release of the RNA within the promoter region

**Fig 10:**
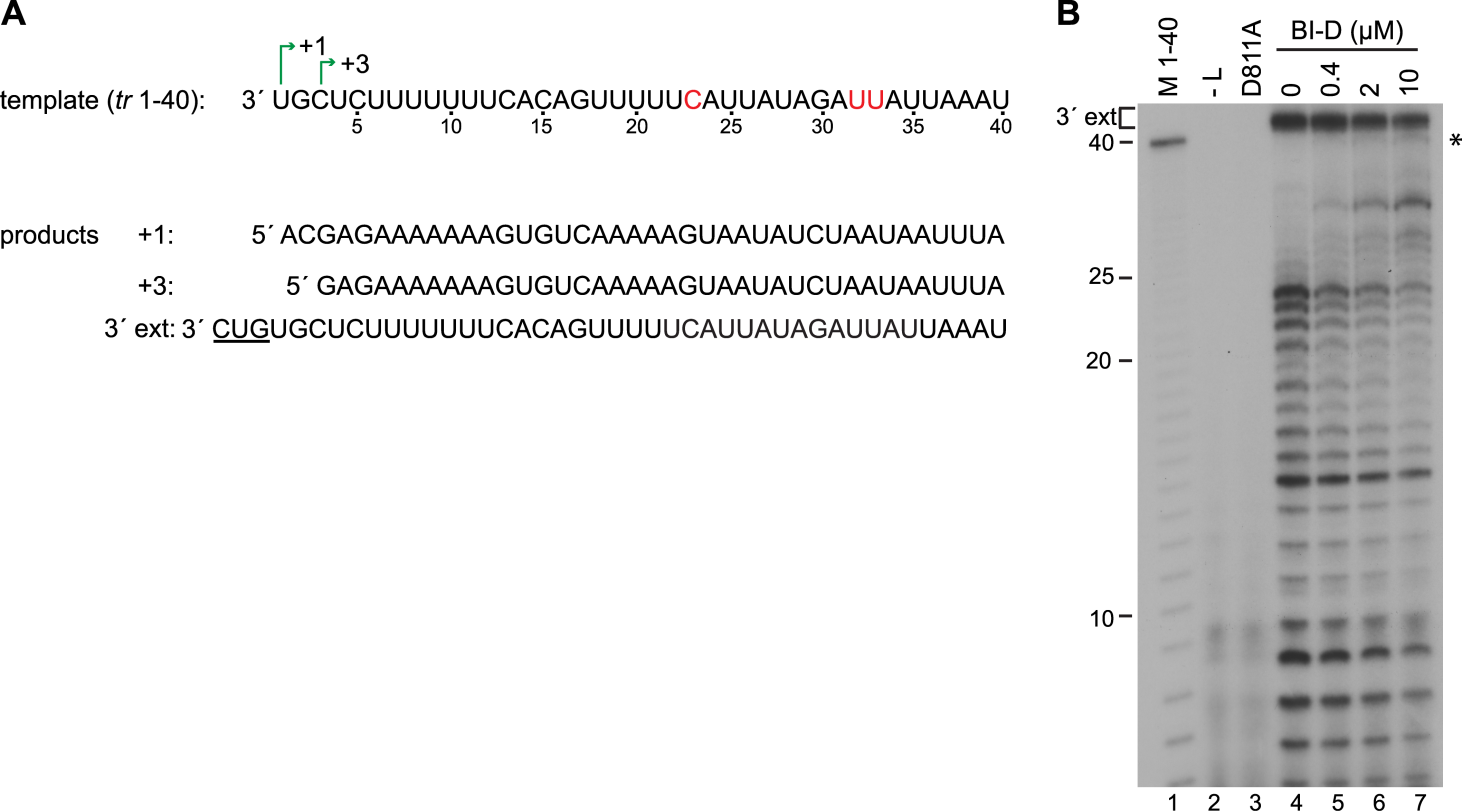
BI-D increased transcription elongation *in vitro*. (A) Sequence of the *tr* 1–40 template used to reconstitute RNA synthesis *in vitro*, with the +1 and +3 initiation sites indicated. Positions 23, 32, and 33 (in red) relative to the 3' end had single nucleotide substitutions to avoid secondary structure within the template. The full-length products that could potentially be generated from this template are shown below and include *de novo* RNA synthesis products initiated at +1 and +3, and the product of 3' extension of the *tr* 1–40 template RNA by back-priming. The nucleotides added by 3' extension are underlined. (B) RNA products generated by wt L-P complexes in the presence of increasing concentrations of BI-D, as indicated. The RNAs were migrated on a 20% urea-acrylamide gel. Lane 1 shows a molecular weight ladder consisting of [γ-^32^P] labeled 40 nt RNA that had been subjected to alkali digestion. Lanes 2 and 3 show negative control reactions performed with either L-P complexes omitted (lane 2), or the catalytically inactive D811A L protein (lane 3). The full length products are indicated with an asterisk.

**Fig 11:**
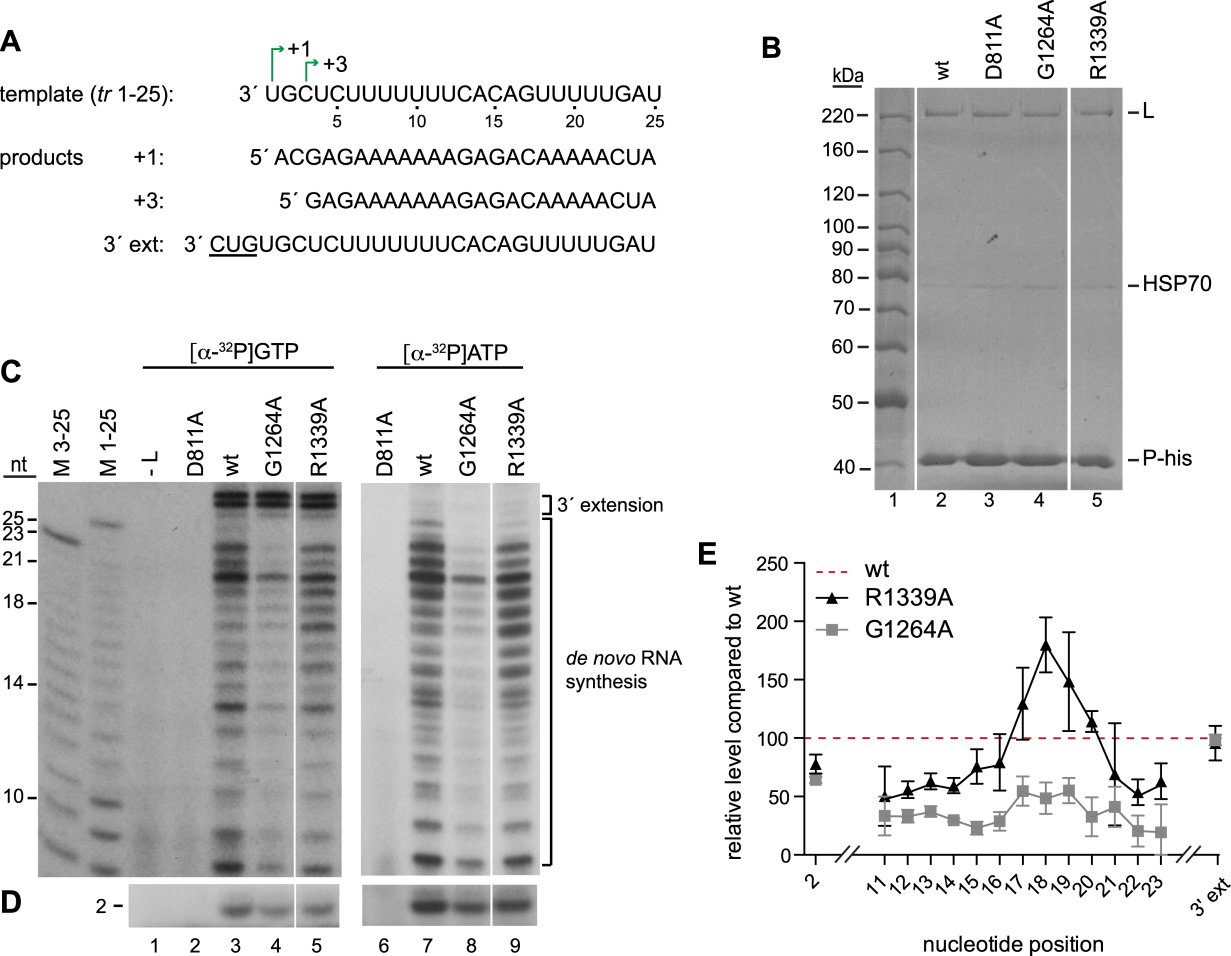
CRV substitutions inhibited polymerase elongation within the promoter. (A) Sequence of the *tr* 1–25 template used to reconstitute RNA synthesis *in vitro*, with the +1 and +3 initiation sites and products of *de novo* RNA synthesis and 3' extension indicated. (B) Analysis of the isolated L-P complexes used for *in vitro* RNA synthesis assays. Colloidal-blue stained SDS-polyacrylamide gel showing isolated wt and variant L-P-his complexes as indicated. The bands corresponding to the expected migration patterns for L, P-his and HSP70, which co-purifies with the complex, are indicated. (C, D) RNA products generated by the wt or variant L-P proteins with either [α-^32^P] GTP or [α-^32^P] ATP were migrated on either a 20% (C) or 25% (D) urea-acrylamide gel adjacent to molecular weight ladders consisting of [γ-^32^P] labeled RNA products corresponding in sequence to the RNA products of the +1 and +3 initiation sites, which had been subjected to alkali digest. Note that because the +1 and +3 products are of different sequence, they migrate slightly differently, particularly in the region from 10–14 nt. Lanes 2 and 6 show negative control reactions performed with the catalytically inactive D811A variant. The white lines on panels B, C and D indicate where lanes on the gel, with samples not described here, were excised. It should be noted that in C, the left and right panels show autoradiograms that were exposed 4 and 3 times longer, respectively than the corresponding panels in D. (E) Quantification of bands from experiments represented by panels C and D. The quantification was performed on data obtained from experiments using [α-^32^P] GTP. The data were normalized to the wt L-P sample, which was set to 100% and is indicated by the red dashed line. The bars show the means and standard deviation of three independent experiments.

The data described above are consistent with the hypothesis that BI-D prevented the polymerase from releasing RNA within the promoter region. However, the data described above from cell-based assays do not exclude the possibility that the truncated transcripts detected on Northern blots arose because BI-D affected the stability of the full-length mRNA and replicative RNAs. Therefore, to confirm if BI-D could cause abnormal elongation of the ~25 nt transcript, RNA synthesis was examined in an *in vitro* RNA synthesis assay using purified recombinant RSV L-P complexes and an RNA oligonucleotide template, as described previously [6] (Fig 10). Because it is challenging to purify the RSV polymerase to a high concentration, we were not able to perform assays in which the initiation and elongation steps were uncoupled, to measure the elongation step of RNA synthesis specifically, neither was it possible to examine the kinetics of elongation. However, it was possible to determine if BI-D affected how far the polymerase could elongate the RNA by analyzing the radiolabeled products using denaturing gel electrophoresis, and then comparing the amounts of shorter and longer transcripts produced in the presence of BI-D, relative to those produced in its absence. The same assay design has been used previously to study how the elongation properties of the VSV polymerase are altered under various conditions [42–44]. RSV polymerase activity was reconstituted with purified L-P complexes, an RNA oligonucleotide consisting of nucleotides 1–40 of the *tr promoter* and NTPs, including [α^32^P]GTP. In previous studies, we have shown that in the *in vitro* assay, the RSV polymerase initiates at positions 1 and 3, as it does in cells, with initiation from +3 being dominant to the extent that the readily detected bands are due to initiation at this position [6, 45] (see also Fig 11). Analysis of RNA generated from the 40 nt template in the absence of BI-D showed two sets of products. At the top of the gel were bands > 40 nt that represent the products of 3' extension (labeled 3'ext.). These arise when the template folds into a panhandle structure, with nucleotides 1 and 2 base pairing with nucleotides 13 and 14 (or 15 and 16), and the polymerase adds up to three nucleotides onto the 3' end of the RNA in a templated fashion, an activity that occurs in infected cells [6]. The level of 3' extension product was not significantly affected by increasing BI-D concentration. The bands ≤ 40 nt represent newly synthesized RNAs that had been initiated at the promoter. In the absence of BI-D the polymerase did not extend these transcripts to the end of the template, but instead paused or released RNAs along the length of template, with the major products being ≤ 25 nt in length (Fig 10B). This correlates with what is found in cells, and shows that the polymerase is not able to efficiently elongate the promoter transcript beyond approximately 25 nt. Titration of BI-D into the reactions did not change the migration patterns of the RNA transcripts ≤ 25 nt (indicating that it did not affect initiation site selection; see legend to Fig 11), but resulted in production of lower amounts of shorter transcripts and the appearance of longer RNA transcripts that were extended to the end of the template (Fig 10B, the asterisk indicates full-length products). This finding shows that BI-D increased the elongation properties of the polymerase and allowed it to overcome the trigger that normally causes release of the RNA after approximately ~25 nt.

### Substitutions in CRV inhibited elongation in the promoter region

We used a similar approach to examine if CRV variant polymerases had elongation defects within the promoter. For this analysis, we focused on two of the L variants which showed different deficiencies in the minigenome system: G1264A and R1339A. G1264A was capable of initiating mRNA synthesis at the *gs* signal, albeit at a lower level than wt polymerase, indicating that it was capable of RNA synthesis (Fig 3). However, it was so deficient in RNA synthesis within the promoter region that RNA initiated from positions 1 and 3 was barely detectable by primer extension analysis, even with the primer that hybridized at positions 13–35 relative to the 5' end of the replicative RNA (Fig 5). In contrast, R1339A was capable of producing RNAs that could be detected with the 13–35 primer, but similarly to G1264A, was defective in producing full-length replication product (Figs 4 and 5). The polymerase complexes containing these variant L proteins were expressed and purified and compared by colloidal blue staining to ensure that similar the preparations contained similar amounts of L and P proteins (Fig 11B). To examine their abilities to elongate RNA within the promoter region, we used a template consisting of the first 25 nt of the *tr promoter* (Fig 11A), as this is the longest distance that the wt polymerase can synthesize RNA (in the absence of BI-D) and is more efficient than the 1–40 template (probably because it is less prone to adopting secondary structures). Previously, we have shown that the polymerase tends to abort RNA synthesis after formation of the first dinucleotide [46]. Therefore, the RNA products from the same RNA synthesis reactions were analyzed using two different gel electrophoresis conditions that were optimized for resolving either the first dinucleotide initiated at position 3 (Fig 11D), or 11–25 nt products, which would be a mixture of RNAs initiated at positions 1 and 3, with the +3 products being dominant, as described above (and being up to 23 nt in length) (Fig 11C). The background in the region of the gel which resolved RNA 3–10 nt in length was typically too high to clearly detect these products. Nonetheless, comparison of the levels of each of the 2 and 11–23 nt products generated by the variants to those generated by the wt polymerase allowed us to gain information regarding how efficiently the variants elongated the RNA relative to the wt polymerase (Fig 11E).

This analysis showed that the R1339A substitution caused subtle, but consistent, differences in the elongation properties of the polymerase, resulting in increased accumulation of products of 17–20 nucleotides, relative to the wt polymerase, and reduced accumulation of products of 21–23 nt (Fig 11C, compare lanes 3 and 5, and 7 and 9; 11E, note that the red dotted line represents the level of each RNA produced by the wt polymerase). This finding shows that the R1339A variant inhibited elongation, resulting in increased accumulation of shorter RNAs. Although the G1264A variant produced dinucleotide at a similar level as the R1339A variant, it produced lower levels of longer RNAs, indicating that this variant was even more deficient in elongation (Fig 11C and 11E). The 3' extension products could be detected in reactions containing [α^32^P]GTP and were similar for each of the variants (Fig 11C, lanes 3–5, 11E). This result shows that all the variants were competent for RNA synthesis *per se*, and that CRV has relatively little impact on the 3' extension activity of the polymerase. However, the G1264A and R1339A substitutions inhibited elongation within the promoter region. The variants might also have been altered in their abilities to initiate RNA synthesis, but we were unable to determine if this was the case because the inability to detect the 3–10 nt products above a high background signal meant that we could not quantify total RNA synthesis relative to the wt polymerase.

## Discussion

The goal of this study was to determine if CRV of the L protein plays a role in RNA synthesis in addition to its anticipated role in mRNA capping. We show that this is the case, and that CRV influences polymerase elongation within the promoter region, with substitutions in CRV reducing elongation distance, and BI-D increasing it.

Based on the previous characterization of the VSV PRNTase domain, and the fact that it is highly conserved between different nsNSVs, it was anticipated that at least some of the substitutions introduced into RSV L in this study would inhibit mRNA capping. Consistent with this, we found that polymerase variants with substitutions at G1264, N1335 and R1339, and to a lesser extent, T1267 and E1269, were deficient in mRNA transcription (Fig 2). Primer extension analysis showed that these variants were capable of initiating RNA synthesis at the *gs* signal, but produced transcripts that either lacked a cap, or that were capped inefficiently (Fig 3). Thus, it appears that the enzymatic domain for PRNTase activity is shared between the rhabdo- and pneumoviruses, although there are some subtle differences, for example with respect to the significance of residue T1267, which is essential for capping in VSV [24, 30].

Single amino substitutions within CRV revealed that this region is also involved in regulating polymerase elongation. Analysis of the G1264A and R1339A variants in an *in vitro* RNA synthesis assay showed that they were less efficient than the wt polymerase at elongating RNA through the promoter region (Fig 11). This is consistent with primer extension analysis of RNAs produced in the minigenome system, which showed that for most of the variants, replication products could be detected with a primer proximal to the 5' end of the RNA (primer 13–35), but not a primer that hybridized further from the 5' end (primer 24–48) (Fig 5). In a Sendai virus polymerase study, double or triple amino acid substitutions were introduced into the CRV [47]. Analysis of the phenotypes of these variants showed that in some cases, they were able to produce abundant levels of le RNA, but could not produce full length replication product [47], similarly to what is described here. This suggests that a role of CRV in regulating elongation during the initial stages of replication may be a common feature of nsNSVs.

Studies with BI-D showed that this compound also affected polymerase elongation through the promoter region. There are three possible mechanisms by which BI-D affected elongation: (i) it could have bound CRV directly, affecting CRV function, (ii) it could have bound elsewhere on the polymerase and allosterically altered the function of CRV, or (iii) it could have bound to CRV and elicited a long-range allosteric effect on the polymerase, which affected elongation. It might be necessary to resolve high-resolution structures of the RSV polymerase, both in isolation and in complex with BI-D, to distinguish between these possibilities. However, based on the fact that substitutions in CRV also affect polymerase elongation we propose that BI-D is most likely exerting an influence by either directly or indirectly altering the properties of CRV. However, whereas amino acid substitutions in CRV caused the polymerase to elongate less efficiently through the promoter, BI-D had the opposite effect, causing the polymerase to be abnormally efficient in elongation through the promoter. Analysis of RNA synthesis *in vitro* showed that whereas the polymerase typically could not elongate beyond ~ 25 nt, in the presence of BI-D this release signal was overcome, resulting in longer RNAs (Fig 10). This finding was consistent with what was detected in the minigenome assay. In this assay, BI-D prevented the polymerase from releasing the small le and tr transcripts after ~25 nt. In the case of the transcription-competent minigenome, this caused the polymerase to read into the first gene instead (Fig 7B and 7H, and Fig 9D) and inhibited mRNA initiation at the first *gs* signal (Fig 7B). This is expected as the polymerase should only be able to reinitiate at the *gs* signal if it had previously released the le transcript.

It has previously been shown (and confirmed here) that failure to cap the 5' end of an mRNA results in abortive RNA synthesis after ~40 nt [24, 32, 33]. It is thought that this is due to a checkpoint at this point in mRNA synthesis such that the polymerase can only elongate the RNA beyond ~40 nt if it can detect a cap. Thus, a role for CRV in elongation had already been suggested. However, the defect in elongation through the promoter region, described here is distinct from that which comes into play at the cap checkpoint. Data from the minigenome system showed that the T1267A variant was partially active in transcription and could produce full-length mRNAs, but was completely defective in replication elongation, whereas the converse was true for the N1335A variant (Figs 2 and 4). This indicates that the capping checkpoint and elongation within the promoter can be separated by single amino acid substitutions. Thus, the data presented here describe a novel function for CRV, namely to modulate elongation within the promoters so that the polymerase is neither hypo- nor hyper-processive. This might be of particular importance in the promoter regions, in which the polymerase must be particularly sensitive to a number of signals to be able to transcribe and replicate the genome. The mechanism by which CRV affects elongation is not known. Structural analysis of VSV polymerase indicates that the lobe containing CRV must undergo a conformational change and move away from the polymerization active site following initiation of RNA synthesis, to allow RNA egress [34]. It is possible that this lobe remains in contact with the RNA to modulate polymerase elongation. CRV could affect polymerase elongation distance by altering a number of factors, including elongation rate (nt polymerized per unit time), and template and transcript affinity. Further experiments will be required to determine which of these factors is influenced by CRV.

While the main finding of this paper is that CRV plays a role in polymerase elongation, the data obtained also indicate that this region has a role in initiation of RNA synthesis. For example, in the case of the minigenome with a *le promoter*, BI-D caused an apparent increase in initiation from +3 (Fig 7; although as noted above, this could be due to more efficient elongation facilitating primer binding), and in the case of the replication-specific minigenome that contained the *tr promoter* at the 3' end, BI-D inhibited initiation at +1 (Fig 9). A role for CRV in initiation is not surprising given that in a pre-initiation state of L, a loop within this region protrudes into the polymerization active site (Fig 1C) [34]. Further studies are underway to more completely characterize the effect of CRV perturbations on initiation at the +1 and +3 sites, and to understand why there appears to be a promoter-specific effect.

The data described above indicate how aberrant elongation induced by BI-D inhibited transcription by preventing the polymerase from being able to reinitiate at the first *gs* signal (Fig 7). They also indicate how the CRV substitutions inhibited replication, as inhibition of elongation within the promoter would not allow replication to occur (Fig 5). However, it is not clear exactly how BI-D inhibited RNA replication. In the case of the *tr promoter*, inhibition of initiation at +1 could be at least partially responsible (Fig 9), but in the case of the *le promoter*, BI-D had little effect on +1 initiation (Fig 7), and increased elongation caused by BI-D might have been expected to facilitate replication, rather than inhibit it. We speculate that BI-D inhibited replication by interfering, either directly or indirectly, with encapsidation. There are two models for encapsidation initiation in the nsNSVs. One is that the 5' end of the replicative RNA contains a sequence that binds to the N protein, thus nucleating the encapsidation process. The other model is that a replicase form of the polymerase binds to N and delivers it to the nascent replicative RNA. These two models suggest two possible mechanisms by which BI-D could inhibit replication. First, it is possible that polymerase elongation through the promoter region must be appropriately calibrated for encapsidation to occur and that BI-D interferes with that. Studies with human RNA-polymerase II have shown that the polymerase pauses after generating a 20–65 nt RNA to allow the recruitment of factors that can modulate gene expression [48]. It is possible that the RSV polymerase must pause in a similar fashion shortly after beginning RNA synthesis, to allow recruitment of N protein to begin the encapsidation process, and that by altering the elongation properties of the polymerase, BI-D prevents this, resulting in production of unencapsidated RNA. The second possibility is that CRV harbors an N binding site that is essential for encapsidation. If this were the case, BI-D might inhibit encapsidation by occluding this site, again resulting in production of unencapsidated RNA. If CRV were to contain an N binding site, this could explain why the R1339A substitution was so deleterious to replication, despite having relatively subtle effects on elongation in the *in vitro* assay.

The results shown here demonstrate that BI-D has pleiotropic effects on the RSV polymerase. However, it is worth noting that the effects of BI-D on abortive transcript production described here differ from what was found in a previous study to examine the mechanism of action of the BI inhibitors [25]. In that study, the mechanism of action of BI-D was not examined, but BI-A and BI-E (compounds related to BI-D) were studied. BI-A caused accumulation of transcripts less than 50 nt in length, and BI-E caused accumulation of transcripts with a 5' triphosphate instead of a 5' cap [25]. Therefore, it was thought that this family of compounds inhibits capping. The results presented here are consistent with this: BI-D inhibited production of capped RNA (Fig 7), however, it resulted in production of abortive transcripts longer than those described previously (Fig 7J). At this time, it is not clear if this is due to differences in the effects of BI-A versus BI-D on elongation, or if it is a reflection of the assays or concentrations of compounds that were used. For example, the assay used in the study by Liuzzi and coworkers used RSV infected cell fractions enriched for viral nucleocapsids [25, 49]. While this assay had the advantage of examining polymerase activity on an encapsidated template, a significant drawback is that it did not allow the researchers to determine the provenance of the RNAs produced. Therefore, it is not known if the abortive transcripts detected in that study were derived predominantly from polymerase that had initiated at one of the viral promoter regions, or from the ten *gs* signals (by polymerases already engaged on the template). The differences between the RNA sequences within these regions, and between the RSV genome and minigenome, could potentially affect polymerase elongation in the presence of compound.

It was already appreciated that CRV of L would be a good target for antiviral drugs. Cap addition in the nsNSVs involves an enzymatically distinct mechanism than cellular capping, and cap addition is essential for viral gene expression [24–26, 29, 32, 50]. The data presented here show that compounds targeting CRV might have the additional benefit of inhibiting replication, which could reduce the possibility of resistance mutations arising. Both defects would result in increased production of RNAs containing a 5' triphosphate, resulting in activation of RIG-I and augmentation of the interferon response, as shown previously [35]. Thus, the findings presented here add further support to the advantages of targeting CRV of L as a strategy for developing antiviral drugs.

## Methods

### Cells

HEp-2 cells (ATCC) were used to confirm expression of the mutant L proteins and were grown in Opti-MEM reduced serum media supplemented with 2% fetal bovine serum (Invitrogen). BSR-T7 cells, that are engineered to constitutively express T7 RNA polymerase [51], were used to reconstitute RSV RNA synthesis using a minigenome template. BSR-T7 cells were grown in G-MEM supplemented with 10% fetal bovine serum, 1X MEM non-essential amino acids solution, 6 mM L-glutamine and 1 mg/ml geneticin (Invitrogen). Sf21 insect cells were used for expression of the RSV L-P complex for assaying RSV RNA synthesis *in vitro*. These cells were cultured in suspension in SF-900 II serum free medium (Invitrogen).

### L plasmid design and mutagenesis

Three types of plasmid expression constructs were generated. To confirm mutant L proteins were expressed in mammalian cells, a codon optimized version of the L ORF of RSV strain A2 containing an N-terminal hexahistidine tag sequence was cloned under the control of the T7 polymerase promoter in pTM1. Mutations in the GDNQ motif of L (D811A) and in CRV (G1264A, T1267A, E1269A, S1333A, N1335A and R1339A) were generated by QuickChange site-directed mutagenesis (Agilent Technologies) in a fragment of the L ORF subcloned into a pGEM T easy vector (Promega). The mutated ORF fragment was sequenced and then substituted in the L ORF of pTM1 by restriction digest and ligation. For analysis of L protein function in the minigenome system, a similar panel of plasmids was generated, lacking the hexahistidine tag. For high-level baculovirus expression of an L-P complex containing substitutions in the GDNQ motif of L (D811A) and in CRV (G1264A and R1339A), CRV mutations were introduced into an L ORF contained in a pFastBac Dual vector. The vector also contained the RSV A2 P ORF tagged with the tobacco etch virus (TEV) protease cleavage site and a hexahistidine sequence, as described previously [6]. Bacmids and recombinant baculoviruses were generated from the pFastBac Dual vectors using the Bac-to-Bac system (Invitrogen), according to the manufacturer’s instructions. All DNA clones were sequenced to confirm the presence of mutations, and to ensure that no other changes were introduced during PCR-mediated mutagenesis.

### Confirmation of variant L protein expression in mammalian cells

To confirm that each of the variant L proteins could be expressed in mammalian cells, HEp-2 cells were transfected with pTM1 plasmids containing histidine-tagged versions of wt or variant L plasmids. Cells were transfected with 0.5 μg of pTM1 P and 0.25 μg wt or mutant L using lipofectin (Invitrogen). Cells were simultaneously infected with modified vaccinia virus Ankara-T7 (MVA-T7) to express the T7 polymerase [52]. The cells were incubated at 37°C and at 48 h post-transfection, they were collected and disrupted with lysis buffer (750 mM NaCl, 50 mM NaH_2_PO_4_, 20 mM imidazole, 0.5% NP40). The cell lysates were clarified by brief centrifugation in a bench top centrifuge and incubated with Ni-NTA agarose (Invitrogen) with gentle rotation for 2h at 4°C The beads were pelleted by centrifugation and after two washes with lysis buffer, the Ni-NTA agarose was pelleted and resuspended in 2X SDS sample buffer. Proteins were analyzed by 10% SDS-PAGE and colloidal blue staining.

### Reconstitution of minigenome replication and transcription in cells

Polymerase activities were assessed in the minigenome system using pTM1 vectors containing untagged versions of the L proteins. The minigenome templates that were used have been described previously [7, 21]. Minigenome RNA synthesis and protein expression were reconstituted in BSR-T7 cells at 32°C, as described previously [20], note that the cells were not co-infected with vaccinia virus or treated with actinomycin D. For samples treated with BI-D, BI-D was diluted in DMSO and added to the media at the time of transfection at the concentrations indicated. DMSO was maintained at a consistent concentration in each reaction. At 48 h post-transfection, cells were harvested to isolate total intracellular RNA. RNA samples were prepared using Trizol according to the manufacturer’s instructions (Invitrogen).

### Northern blot analysis

To detect full length mRNA and replication products generated from minigenomes, RNA samples were subjected to electrophoresis in 1.5% agarose-formaldehyde gels in MOPS buffer and subjected to Northern blot analysis as previously described [7]. The identities of each of the bands were determined by comparing the RNAs produced by minigenomes that have different arrangements of *gs* and *ge* sequences, and by examining RNAs with gene specific probes (e.g. as described in [13]). In each experiment, the levels of input minigenome RNA were determined by probing Northern blots with a positive sense CAT riboprobe. To detect low molecular weight RNAs, RNA was analyzed by gel electrophoresis in 6% polyacrylamide gels containing 7 M urea in Tris-borate-EDTA buffer. The ladders used were either a low-range ssRNA ladder (NEB), which was excised from the gel prior to transfer, stained with ethidium bromide for visualization with UV light and then realigned with the Northern blot, or the Decade Marker System (Ambion) which was Northern blotted directly. Direct comparison showed correlation between the two markers. Northern Blots were analyzed by autoradiography and phosphorimager analysis.

### Primer extension analysis

5'-ends of the RNA transcription and replication products were analyzed by primer extension as described previously [7, 20, 21]. In addition, a primer that hybridized at position 91–113 of the positive sense RNA products was used (5' TATCCAGTGATTTTTTTCTCCAT). RNA samples were reverse transcribed at 37°C using the Sensiscript RT kit (Qiagen) and radiolabeled primers. To detect capped RNA, reverse transcription reactions were performed using Thermoscript reverse transcriptase (Invitrogen) at 55°C. In cases in which the guanosine cap of transcription products were digested with pyrophosphatase, RNA was treated with Cap-Clip Acid Pyrophosphatase (CellScript) for 2 h at 37°C prior to primer extension. Primer extension reactions were analyzed by electrophoresis in 6% or 8% polyacrylamide gels containing 7 M urea in Tris-borate-EDTA buffer. End-labeled oligonucleotides corresponding in sequence to cDNA representing initiation from +1 and +3 of the *le* and *tr promoters* and the first position of the *gs* signal were used as markers. Primer extension reactions were analyzed by autoradiography and phosphorimager analysis.

### *In vitro* RNA synthesis assays

The RSV L and P proteins were expressed in insect cells using recombinant baculovirus and the L-P complex was purified as described previously [6], except that the proteins were eluted from Ni-NTA beads with sodium phosphate buffer containing 250 mM imidazole and then dialyzed into 150 mM NaCl, 20 mM Tris HCl pH 7.4, 10% glycerol, 1 mM DTT. To compare wt and variant polymerases, polymerase preparations were tested for RNase contamination by incubating them for 30 minutes, 1 h, and 2 h in a reaction buffer of the same composition used for RNA synthesis assays, with an end labeled RNA corresponding in sequence to the product of the 25 nt template, and the products were analyzed by denaturing gel electrophoresis, as described below. Polymerase preparations showing evidence of nuclease contamination were not included in the results presented here.

RNA synthesis was reconstituted *in vitro* using a similar approach as described previously [6, 7], but with some modifications. A PAGE-purified RNA oligonucleotide corresponding to nucleotides 1–25 or 1–40 of the *tr promoter* was used as a template. Positions 23, 32, and 33 relative to the 3' end of the 1–40 template were changed to C, U, and U respectively to limit secondary structure. RNA (2 μM) was combined with the purified L-P complex (containing ~200 ng of L protein) in RNA synthesis buffer (50 mM Tris HCl pH 7.4, 8 mM MgCl_2_, 5 mM DTT, 10% glycerol), with 500 μM rNTPs, and 10 μCi of [α-^32^P] GTP or [α-^32^P] ATP in a final volume of 50 μl. For reactions containing BI-D, BI-D was diluted in DMSO and added to reactions at the concentrations indicated. DMSO was maintained at a consistent concentration in each reaction. Reactions were incubated at 30°C for 2 h, followed by incubation at 90°C for 3 minutes to inactivate the polymerase, and then diluted in an equal volume of stop buffer (deionized formamide containing 20 mM EDTA, bromophenol blue, xylene cyanol). To resolve 2 nt products, the reactions were analyzed on a 25% polyacrylamide gel containing 7 M urea in Tris-taurine-EDTA buffer. Longer RNA products were resolved by electrophoresis on a 20% polyacrylamide gel containing 7 M urea in Tris-borate-EDTA buffer. The nucleotide lengths of the RNA products were determined by comparison with a molecular weight ladder generated by alkaline hydrolysis of [γ^32^P] ATP end-labeled RNA oligonucleotides representing the anticipated 25 and 23 nt RNA products produced from the +1 and +3 sites, respectively or by alkaline hydrolysis of [γ^32^P] ATP end-labeled 1–40 RNA. Bands were visualized by autoradiography and quantified using Licor Image Studio Lite.
